# Exploring the feasibility of real-time on-device ECG biometric classification using quantized neural networks

**DOI:** 10.3389/fdgth.2025.1626279

**Published:** 2026-02-03

**Authors:** Martin Berki, Anton Mateasik, Michal Micjan, Erik Vavrinsky, Krisztian Gasparek, Lubos Cernaj

**Affiliations:** 1 Institute of Electronics and Photonics, Slovak University of Technology, Bratislava, Slovakia; 2 International Laser Centre, Bratislava, Slovakia

**Keywords:** electrocardiogram, embedded systems, neural networks, Biometric Identification, Quantized Inference

## Abstract

Biometric classification using electrocardiogram (ECG) signals offers a promising pathway for continuous, personalized healthcare monitoring. This work presents a proof-of-concept embedded deep learning system for real-time ECG biometric classification on wearable Holter devices, reducing reliance on continuous cloud connectivity. A quantized convolutional neural network (CNN) was deployed on an STM32H7 microcontroller to identify individuals based on unique ECG patterns, incorporating an initial signal quality assessment stage to ensure that only high-quality segments are processed. Evaluated on the PTB Diagnostic ECG Database with subject-specific training, the system achieved F1 score of 94.51% and a classification accuracy of 94.68% on five-second ECG segments, with an average inference time of 1.35 s, enabling real-time operation on resource-constrained hardware. By performing on-device inference, the system improves data privacy, can reduce power consumption, and minimizes unnecessary data transmission. This embedded implementation demonstrates the feasibility of integrating lightweight ECG biometrics into wearable systems, with potential for future extensions toward personalized healthcare monitoring and early anomaly detection.

## 1 Introduction

Cardiovascular diseases (CVDs) continue to represent the leading cause of mortality worldwide, accounting for approximately 20.5 million deaths in 2021, which constitutes nearly one-third of all global fatalities [1]. In the European Union alone, CVDs were responsible for 32.4% of deaths during the same period [2]. Early detection, effective self-care, and continuous monitoring are critical strategies for mitigating complications and improving long-term patient outcomes [3, 4].

Electrocardiogram (ECG) continues to play a central role in first-line cardiac diagnostics, providing a non-invasive method to evaluate the heart’s electrical activity [5]. Standard 12-lead ECG recordings, typically performed during scheduled clinical check-ups [6], offer a comprehensive but transient snapshot of cardiac function. However, for patients with chronic heart conditions or those at elevated risk of ECG-detectable abnormalities, isolated measurements are insufficient to capture intermittent or evolving pathologies [7].

To address this limitation, portable ECG devices such as Holter monitors have been widely adopted to facilitate continuous cardiac monitoring over periods ranging from 24 h to several weeks [8]. Holter recordings provide valuable longitudinal data, capturing transient arrhythmias and anomalies that would otherwise go undetected during brief clinical assessments [9]. While long-duration ECG monitoring offers new opportunities, it also introduces specific challenges. Extended recordings generate massive volumes of data, which, while rich in diagnostic potential, are computationally demanding to store, process, and analyze [10, 11].

Historically, Holter monitors have operated as standalone systems, recording data locally for offline analysis [12]. Recent advances, however, have driven the evolution toward continuous wireless transmission of ECG data using wearable patches, smartwatches, and next-generation Holter devices [13, 14]. These cloud-connected systems enable near real-time analysis, often powered by artificial intelligence (AI) algorithms, providing scalable solutions for automated diagnostics and long-term health monitoring [15, 16].

Despite these benefits, cloud-based transmission introduces substantial trade-offs. Continuous data streaming requires significant bandwidth, increases power consumption, and raises concerns regarding data privacy and security. Moreover, reliance on network connectivity can lead to data transmission delays and intermittent loss of service, causing local storage bottlenecks during disruptions [16–18]. These constraints highlight the need for more efficient data handling strategies in wearable cardiac monitoring systems.

Optimizing the efficiency of continuous ECG monitoring requires the ability to preprocess and classify signals directly on the device. By identifying and discarding low-quality or clinically irrelevant segments at the source, it can help reduce transmission loads, potentially extend battery life, and enhance user privacy. Embedded artificial intelligence offers a promising approach to achieving this goal, enabling real-time, on-device ECG analysis that reduces reliance on external computational infrastructure.

### 1.1 The role of AI in personalized cardiac monitoring

Artificial intelligence has had a transformative impact on healthcare, enabling advancements in automated diagnostics, pathology classification, and clinical decision support systems [19–21]. In the context of cardiology, AI-driven analysis of ECG signals has achieved significant success in the detection of arrhythmias, myocardial infarctions, and other cardiac abnormalities [22–27]. However, the majority of current AI applications rely on cloud-based infrastructures, requiring stable network connectivity and substantial computational resources to operate effectively [28–30].

Beyond conventional population-wide models, an emerging area of research focuses on personalized AI systems that adapt to the unique physiological characteristics of individual patients. Unlike generic AI models trained on large, heterogeneous datasets, personalized models learn a specific patient’s baseline ECG patterns, enabling the detection of subtle deviations that may signify early pathological changes. This individualized approach is particularly valuable for long-term monitoring of patients with progressive cardiac conditions, where gradual shifts in ECG morphology can provide early indicators of deterioration [31–34].

By establishing a dynamic, patient-specific understanding of cardiac function, personalized AI offers the potential to move beyond static diagnostics and toward continuous, adaptive health monitoring. Realizing this potential, however, requires solutions capable of operating directly on wearable devices, minimizing reliance on external infrastructure while maintaining real-time responsiveness.

### 1.2 Embedded AI for biometric ECG classification

Biometric classification involves the identification or verification of individuals based on their unique physiological or behavioral traits, such as fingerprints, facial features, or, in this study, electrocardiogram (ECG) signals. Traditional biometric systems extract distinctive features from input data to establish or confirm identity with high confidence [35, 36].

In the context of cardiac monitoring, the morphology of an individual’s ECG-comprising the specific shapes, intervals, and rhythms of each heartbeat-encodes subtle, person-specific electrical patterns. These patterns are influenced by anatomical variations, electrophysiological properties, and electrode placement [37, 38]. By taking advantage of these inherent differences, ECG-based biometric systems can uniquely characterize individuals, offering an alternative to conventional biometric modalities.

This study explores the integration of biometric ECG classification directly into a wearable Holter device, enabling the system to autonomously verify patient identity in real-time without reliance on cloud servers. Rather than diagnosing cardiac abnormalities, the primary objective is to determine whether the captured ECG segment originates from the intended user. This personalized approach supports the establishment of an individual cardiac baseline, forming the foundation for future systems capable of continuous patient-specific health tracking and, eventually, the detection of deviations indicative of physiological deterioration.

## 2 Related work

Research in ECG biometrics has traditionally focused on security and identification applications, where ECG signals are used as unique physiological signatures to verify individual identity. In these approaches, distinctive features such as heartbeat intervals, waveform morphology, and rhythm patterns are extracted to establish identity with high confidence [39–45]. Studies have demonstrated the robustness of ECG-based biometrics across varying sensor types and acquisition conditions, supporting its potential for practical deployment.

Beyond security applications, the integration of ECG biometrics into healthcare diagnostics has recently emerged as a promising research direction. Shusterman and London [46] proposed a personalized ECG monitoring (pECG) framework that combines adaptive machine learning and distributed architectures to refine patient-specific baselines over time. Their system extracts minimal features locally while relying on server-based adaptation to handle evolving physiological changes, illustrating the benefits of personalization in continuous cardiac monitoring.

Similarly, Mangold et al. [47] conducted a large-scale study using over 970,000 ECG records from approximately 100,000 patients to evaluate the feasibility of ECG signals as biometric identifiers. Employing a Siamese neural network architecture, they achieved high matching accuracy and further demonstrated that single-lead ECGs can approach the performance of full 12-lead configurations. Their analysis of longitudinal data also highlighted the dynamic nature of ECG fingerprints, suggesting the importance of adaptive modeling for long-term applications.

Efforts to implement real-time ECG analysis on embedded hardware have also been reported. Raj and Ray [48] developed a microcontroller-based monitoring system capable of local feature extraction and arrhythmia detection. Their approach relied on classical signal processing methods, including the Discrete Cosine Stockwell Transform and support vector machines, to achieve efficient on-device classification with low power consumption.

Recent lightweight 1D CNNs for single-lead ECG have also been explored for arrhythmia detection. Rahman and Faezipour [49] proposed a compact time-domain CNN and argued that its computational footprint makes it suitable for embedded deployment. While their focus is population-level arrhythmia classification rather than biometric verification, the architectural theme—shallow temporal convolutions over short ECG windows—aligns with our motivation for compact models on constrained devices. In contrast, our study emphasizes a fully embedded, subject-specific pipeline with static int8 quantization, explicit memory/timing analysis on a Cortex-M7, and a two-stage flow with signal-quality gating.

Wang et al. [50] proposed an ECG biometric authentication framework based on self-supervised representation learning for IoT edge devices. Their approach employed a convolutional encoder trained via contrastive pre-training on largely unlabeled ECG data, followed by correlation-based identity verification using per-subject templates. Evaluated on the PTB Diagnostic ECG Database and cross-validated on external datasets such as MIT-BIH Arrhythmia and ECG-ID, their method achieved high authentication accuracy (≈ 99.1% on PTB-DB and ≤ 98.5% on unseen datasets), demonstrating strong generalization capabilities. To support embedded operation, they further implemented model pruning and 8-bit quantization on a Cortex-M4F microcontroller, reporting only marginal accuracy loss (0.5%) while reducing computational cost by approximately 37%. This work highlighted the potential of self-supervised feature extraction for low-power biometric systems and emphasized the scalability of global ECG encoders across multiple users. In contrast, the present work pursues a complementary objective: instead of learning a population-wide ECG representation for universal authentication, it investigates a personalized verification paradigm in which the model is trained to recognize a single individual’s cardiac signature directly on the device. The proposed framework integrates a compact, statically quantized CNN with an initial signal-quality assessment stage, enabling selective and fully autonomous operation on the embedded hardware.

Collectively, these studies demonstrate significant progress toward personalized, automated ECG analysis. They highlight the potential of ECG biometrics for continuous health monitoring, the importance of adapting to individual physiological baselines, and the feasibility of deploying classification systems on embedded platforms. However, challenges remain in integrating deep learning-based personalization into fully embedded systems with significantly reduced reliance on cloud infrastructure, particularly under the resource constraints typical of wearable Holter devices.

## 3 Methodology

Real-time ECG biometric classification on embedded hardware was achieved through a structured workflow, addressing the specific challenges of operating within the computational and memory constraints of a resource-limited microcontroller.

### 3.1 Data acquisition and preprocessing

ECG data were sourced from the PTB Diagnostic ECG Database [51]. We used five subjects in total: Subject 180 (7 recordings), Subjects 093 and 233 (5 recordings each), and Subjects 007 and 011 (4 recordings each). Primary evaluation was performed on Subject 180 due to the larger number of available recordings, while multi-subject validation and benchmarking were conducted on Subjects 093, 233, 007, and 011.

To simulate Holter monitoring, only the aVL lead was used. Signals sampled at 1,000 Hz were downsampled to 500 Hz to reduce computational load while preserving morphological features. Data were segmented into non-overlapping 5-s windows and standardized to zero mean and unit variance prior to inference. The standardized 5-s segments were then passed to the signal-quality stage followed by the biometric classifier as described below.

### 3.2 Signal quality assessment

Prior to biometric classification, the system performs an initial signal quality assessment to ensure that only clinically relevant ECG segments are processed. This step is essential, as long-duration Holter recordings often include artifacts caused by motion, poor electrode contact, or environmental noise. To enable automated filtering, a custom dataset of 187,000 manually labeled ECG segments was created, distinguishing usable signals from degraded ones based on visual inspection. A convolutional-recurrent neural network (CNN-LSTM) trained on this dataset achieved an F1 score of 90.06% in separating high-quality segments from noise [52].

Labeling was conducted by two independent groups of trained annotators following a joint briefing and calibration session with shared guidelines. Group A and Group B each annotated one half of the dataset in the first pass, after which the halves were swapped for a second-pass review of the other group’s labels. Samples for which the second pass did not yield consensus were removed from the final dataset.

For embedded deployment, the CNN-LSTM was replaced with a more efficient pure-CNN model to reduce inference time and memory usage on the STM32H7 microcontroller. To further optimize memory usage, the signal quality assessment network was aligned with the biometric classification model, sharing the same architecture and differing only in trained weights. This design enables efficient weight swapping between inference stages, minimizing memory overhead and simplifying deployment while maintaining strong classification performance.

On the held-out signal-quality test set, the pure-CNN achieved an F1 score of approximately 88.2%, representing a drop of about 2 percentage points compared with the CNN-LSTM baseline. This reduction was accepted in exchange for substantially lower latency and memory footprint on the STM32H7.

### 3.3 Neural network architecture

The biometric classifier is a lightweight CNN with four convolutional layers and a fully connected head. The first convolutional layer’s receptive field spans 250 ms, to fully capture QRS complex and adjacent waveforms. The architecture was experimentally optimized to balance accuracy and resource efficiency.

### 3.4 Training procedure

Standard training procedures were used (Adam optimizer, binary cross-entropy loss) for 50 epochs, with training and validation sets drawn from different ECG recordings.

### 3.5 Model quantization and embedded deployment

The trained model was statically quantized to 8-bit precision using ONNX [53], with calibration on a subset of the training data. A two-stage inference system was deployed on the STM32H7 microcontroller: the first pass assesses signal quality, and if acceptable, new model weights are reloaded for biometric classification. Although direct power measurements were not conducted, quantized inference and conditional execution are expected to reduce energy consumption.

## 4 Data

This study utilized the PTB Diagnostic ECG Database [51]. A summary of key database attributes is provided in Table 1. The database contains high-resolution, multi-channel ECG recordings from 290 subjects, sampled at 1,000 Hz with 16-bit resolution, and includes conventional 12-lead recordings alongside Frank leads. Basic demographic and clinical annotations are also provided.

**Table 1:** Attributes of the PTB diagnostic ECG database v1.0.0.

Attribute	Description
Title	PTB Diagnostic ECG Database v1.0.0
URL source	https://www.physionet.org/content/ptbdb/1.0.0/
Number of records	549 high-resolution 15-lead ECGs
Number of subjects	290 subjects, each with 1 to 5 ECG records
Demographics	Ages 17 to 87 (mean age: 57.2 years); 209 males (mean age: 55.5), 81 females (mean age: 61.6)
ECG leads	15 simultaneously measured signals: 12 standard leads (I, II, III, aVR, aVL, aVF, V1, V2, V3, V4, V5, V6) and 3 Frank leads (vx, vy, vz)
Sampling rate	1,000 Hz (with recordings available up to 10 kHz upon request)
Resolution	16-bit digitization with a range of ± 16.384 mV
Input voltage	± 16 mV, compensated offset voltage up to ± 300 mV
Bandwidth	0–1 kHz (synchronous sampling of all channels)
Noise voltage	Maximum 10 μV (peak-to-peak), 3 μV (RMS) with input short circuit
Clinical Annotations	Includes age, gender, diagnosis, medical history, medication, interventions, coronary artery pathology, ventriculography, echocardiography, and hemodynamics (not available for 22 subjects)
Diagnostic classes	Myocardial Infarction: 148
	Cardiomyopathy/Heart Failure: 18
	Bundle Branch Block: 15
	Dysrhythmia: 14
	Myocardial Hypertrophy: 7
	Valvular Heart Disease: 6
	Myocarditis: 4
	Miscellaneous: 4
	Healthy Controls: 52
Data format	Each record includes a header (.hea) file with clinical summaries and a data (.dat) file containing the ECG signals
Access and license	Open Access under the Open Data Commons Attribution License v1.0

For model development and evaluation, we trained and evaluated subject-specific models for five subjects (IDs 180, 093, 233, 007, and 011). Subject 180, a 37-year-old healthy male with seven recordings (each approximately 10 min), served as a primary example due to the larger number of recordings, but all five subjects were included for training, validation, and benchmarking as outlined in the Methodology. This multi-subject setup ensured that training, validation, and testing were conducted across distinct recordings on a per-subject basis.

Only the aVL lead was used to simulate a Holter setup, reflecting the single-lead configuration of our target wearable device. Although data acquisition from our custom Holter system is planned for future work, the PTB data was used to establish initial feasibility. Signals were downsampled from 1,000 Hz to 500 Hz to reduce computational overhead while preserving critical features such as QRS complexes and ST-segment morphology. Each recording was segmented into non-overlapping 5-s windows and standardized to zero mean and unit variance prior to training.

## 5 Target hardware

**Figure 1:**
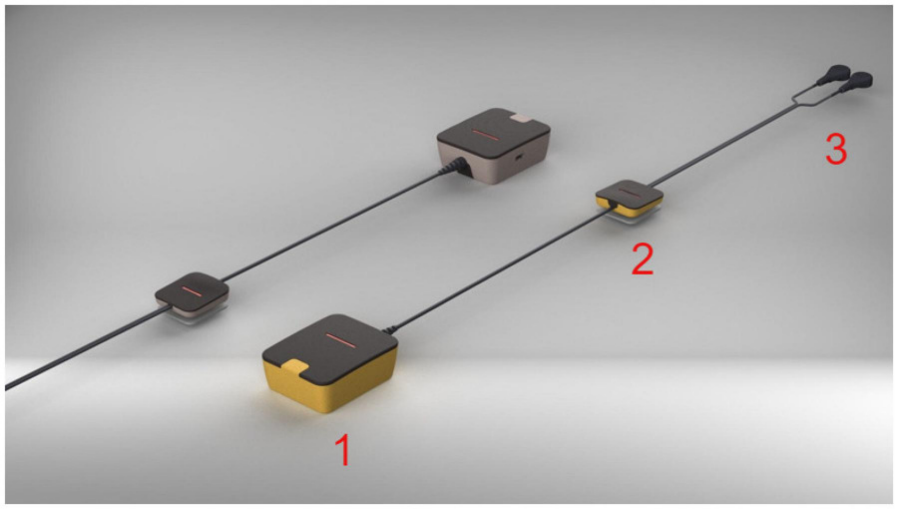
Three-dimensional render of the self-developed single-lead ECG Holter device designed for continuous wearable monitoring. The system consists of three interconnected modules: (1) the main digital/analog module housing the STM32H7 microcontroller, analog front-end, and wireless communication interface; (2) the ground reference electrode providing common-mode noise rejection and patient isolation; and (3) the right-arm (RA) and left-arm (LA) sensing electrodes responsible for ECG signal acquisition. All components are connected through low-profile shielded cables to ensure minimal motion artifacts and user comfort. The modular design enables both high-quality signal acquisition and efficient on-device neural network inference for real-time biometric classification.

The target platform for this study is a custom-developed wearable Holter device designed for single-channel ECG acquisition [54]. The device is depicted in Figure 1. It uses a three-electrode configuration (right arm, left arm, and ground) suitable for continuous ambulatory monitoring. The device integrates a high-performance STM32H743VI6 microcontroller, enabling real-time on-device neural network inference.

Key hardware specifications relevant to embedded AI deployment are summarized in Table 2. The Cortex-M7 processor, combined with 1 MB RAM and efficient DMA support, provides the necessary computational resources for running quantized convolutional models within tight power and memory constraints.

**Table 2:** Key specifications of the STM32H743VI6 microcontroller.

Parameter	Specification
Core	32-bit Arm Cortex-M7, 480 MHz, double-precision FPU, DSP instructions
RAM	1 MB (192 KB TCM + 864 KB SRAM)
Flash memory	2 MB (read-while-write support)
DMA controllers	4 DMA engines, including 1 Master DMA (MDMA)
Clock sources	HSI 64 MHz, HSE 4–48 MHz, LSE 32.768 kHz, 3 PLLs
External memory interface	Dual-mode Quad-SPI, SDRAM, NOR/NAND flash support (up to 100 MHz)

While the device supports modular upgrades such as multi-lead ECG acquisition, auxiliary sensing (IMU, barometer, magnetometer), and 2G/4G cellular communication, these features were not utilized in the present study. The current setup focuses solely on the future single-lead ECG acquisition and processing for proof-of-concept validation.

## 6 Convolutional neural network architecture

**Figure 2:**
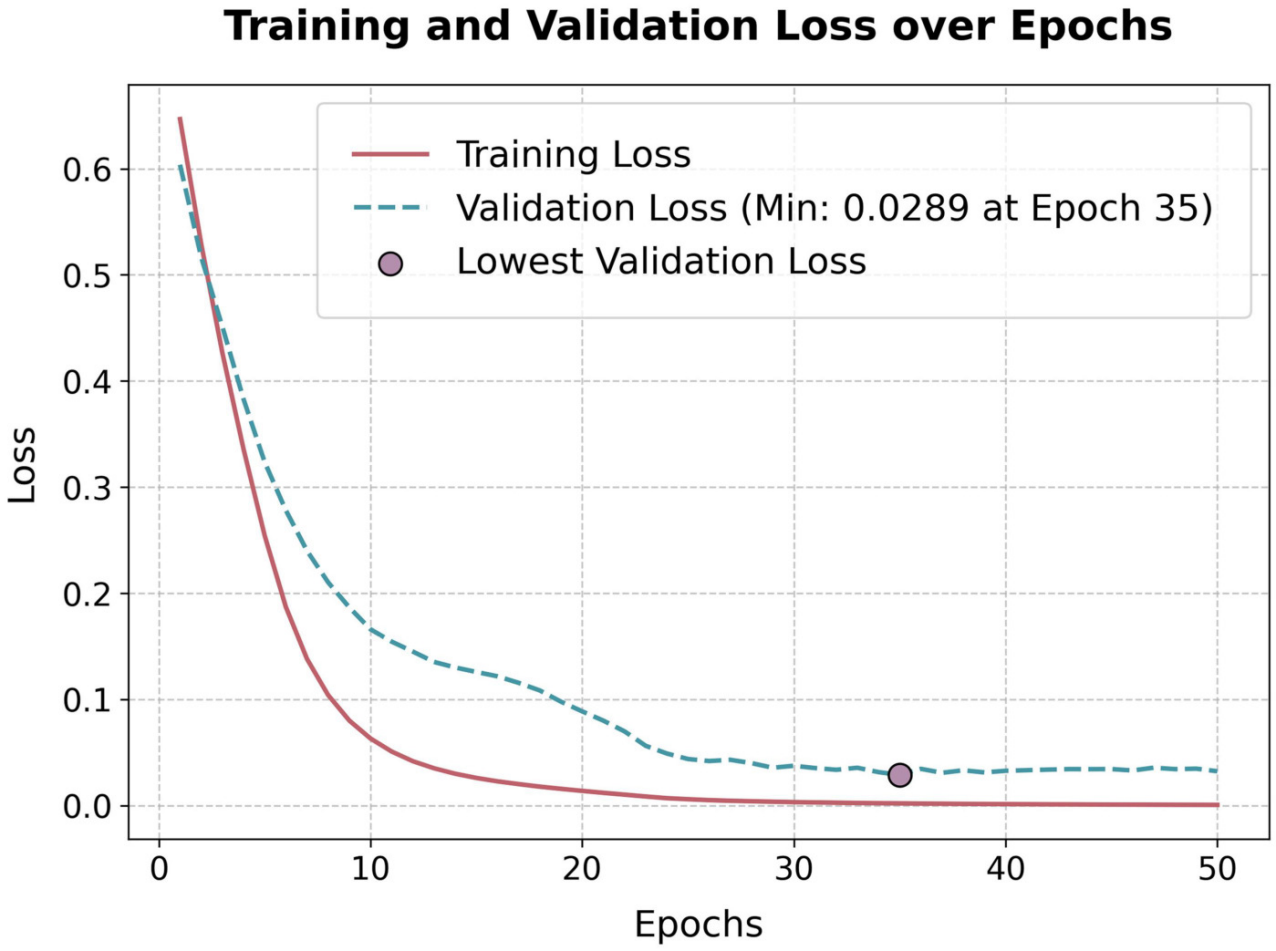
Training and validation loss over 50 epochs for the PTB dataset. The model’s validation loss steadily declined during early epochs, reaching a minimum of **0.0289** at epoch 35.

The proposed convolutional neural network (CNN) processes 5-s, single-channel ECG segments sampled at 500 Hz, corresponding to 2,500 time steps per input. The architecture consists of four convolutional layers followed by a fully connected classifier, progressively transforming raw ECG waveforms into an abstract representation suitable for classification. Although convolutional networks are often treated as ”black boxes,” their hierarchical structure allows them to learn increasingly complex patterns [55], as demonstrated by Figure 2, which visualizes the output of selected filters at different layers.

**Figure 3:**
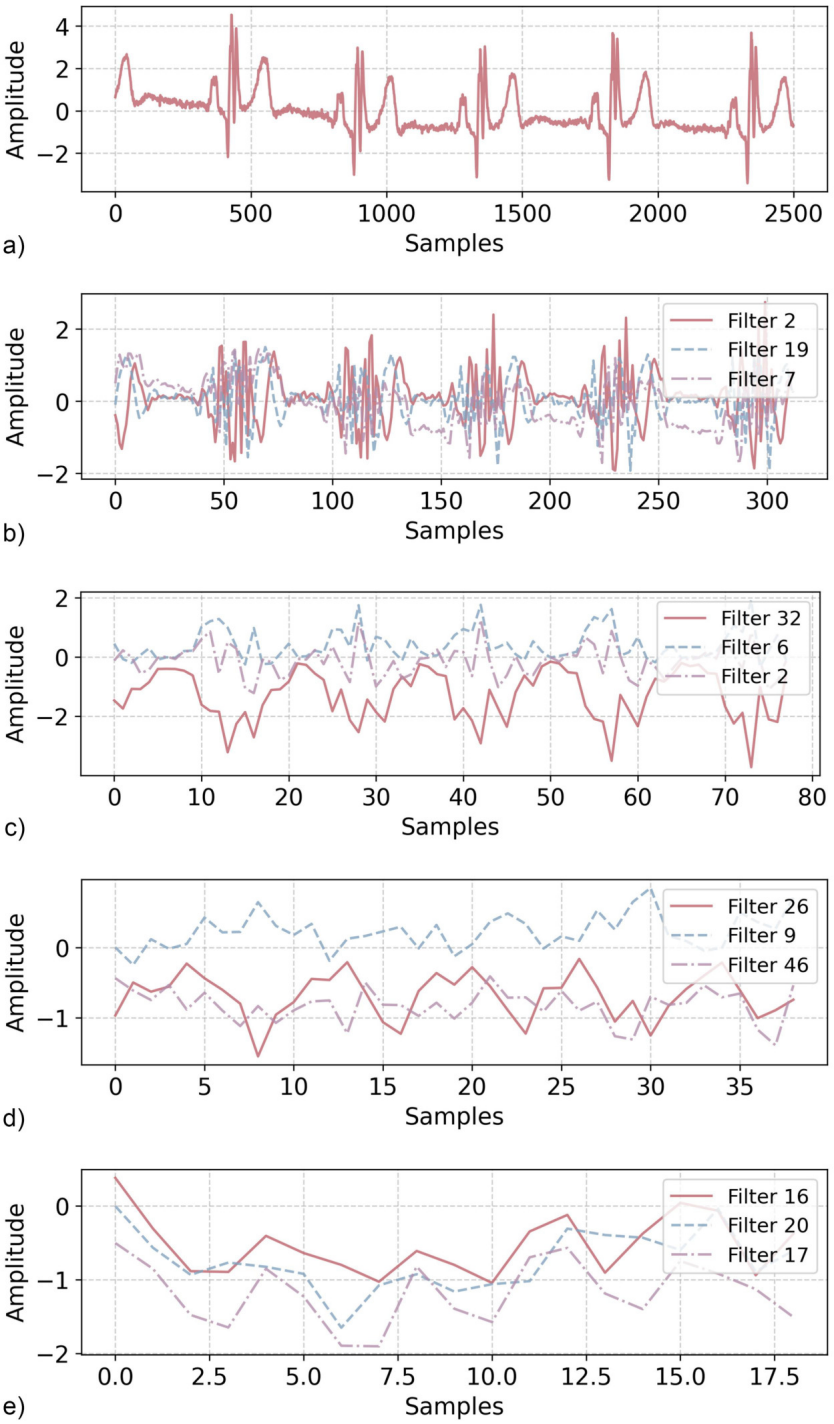
Visualization of convolutional layer outputs. The original ECG signal (top) is gradually transformed as it moves through the network. Early layers keep high-frequency components, while deeper layers capture broader waveform patterns. (**a**) Original signal. (**b**) Convolved signals at Layer 1. (**c**) Convolved signals at Layer 2. (**d**) Convolved signals at Layer 3. (**e**) Convolved signals at Layer 4.

The first convolutional layer applies 32 filters with a kernel size of 125 and a stride of 8, effectively downsampling the signal while preserving its key morphological characteristics. This layer’s receptive field spans 250 ms, allowing it to process an entire QRS complex along with adjacent waveform components. Batch normalization is used to stabilize training, followed by a ReLU activation. A max-pooling operation with a kernel size of 4 and stride 4 further reduces the temporal resolution, outputting a feature map of shape (32, 78), where each feature encodes localized signal variations. As illustrated in Figure 3b, this stage predominantly highlights sharp transitions, which are characteristic of QRS complexes and other high-frequency elements.

The second convolutional layer refines these initial representations using 32 filters with a smaller kernel size of 3, a stride of 1, and padding of 1, ensuring that local signal morphology is preserved while allowing the network to detect subtle waveform variations. Another max-pooling operation (kernel size 2, stride 2) reduces the feature map to (32, 39). At this stage, as seen in Figure 3c, the extracted features still retain noticeable ECG morphology but become more invariant to small perturbations.

The third convolutional layer expands the network’s capacity by increasing the number of filters to 64 while maintaining a kernel size of 3 and a stride of 1. A subsequent max-pooling operation (kernel size 2, stride 2) further reduces the temporal dimension to (64, 19), distilling the most salient inter-beat patterns. Figure 3d demonstrates that this layer begins to focus more on rhythm-based variations rather than fine waveform details, aiding in subject-specific identification.

The final convolutional layer (Figure 3e) preserves the structural integrity of the extracted features while condensing them into a compact representation. Using 64 filters with a kernel size of 3 and an adaptive average pooling operation, it outputs a feature map of size (64,1), which serves as an abstract signature of the input ECG segment. Unlike earlier layers, which retain interpretable waveform-like structures, this stage generates highly distilled feature vectors that no longer resemble the raw input but encapsulate key distinguishing characteristics.

**Figure 4:**
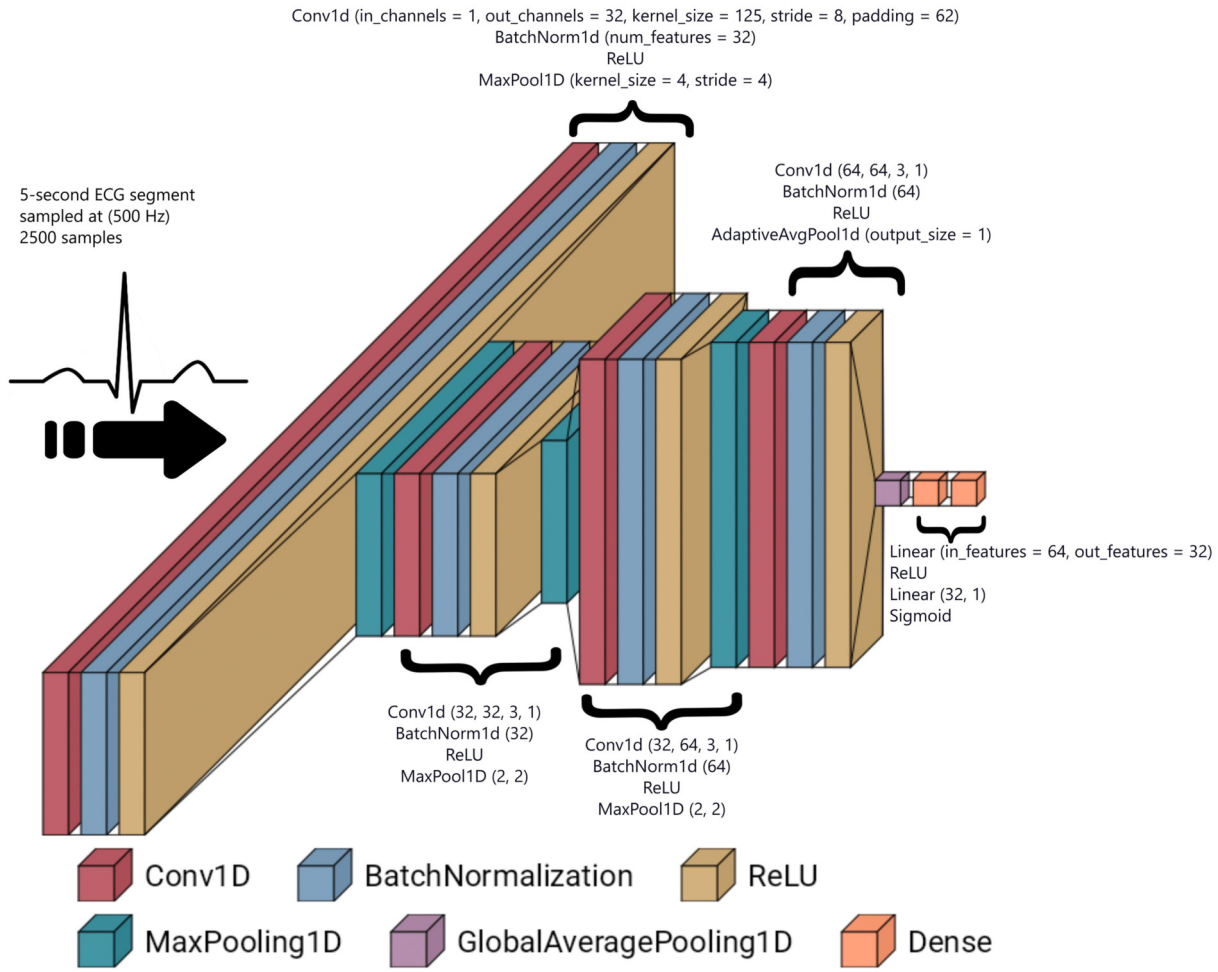
The architecture of the proposed CNN, consisting of four convolutional layers followed by a fully connected classifier. The model gradually transforms ECG signals into feature representations suitable for classification.

Following convolutional feature extraction, the output is flattened and passed through a fully connected classifier. The first dense layer maps the 64-element feature vector to 32 neurons with ReLU activation, followed by a final layer that applies a sigmoid activation function to produce a probability score in the range [0,1], representing the likelihood that the input belongs to a specific individual. The fully connected layers refine the high-level representations, reinforcing subject-specific characteristics while discarding redundant information. Visualization of the network's layers is depicted in Figure 4.

## 7 Training results for biometric classification

We trained and evaluated subject-specific models for five enrolled subjects (180, 093, 233, 007, 011). For each subject, training, validation, and testing sets were drawn from distinct recordings to avoid leakage. For Subject 180—which had seven recordings—we used four recordings for training (≈ 40 min), two for validation, and one for testing. For the remaining subjects, analogous per-subject splits were used; the segment-level Train/Test/Eval counts are summarized in Table 3. In all cases, evaluation used recordings unseen during training and validation.

**Table 3:** Verification performance per enrolled subject.

Patient	Model	Domain	Train/test/eval	PC-F1	PR-AUC
180	CNN	Time	95/48/24	0.95	0.99
	MobileNet	Time		0.88	0.95
	CNN	FFT		0.41	0.69
	MobileNet	FFT		0.44	0.54
	SVM	FFT		0.68	0.86
093	CNN	Time	69/23/23	0.93	0.97
	MobileNet	Time		0.42	0.63
	CNN	FFT		0.69	0.82
	MobileNet	FFT		0.75	0.89
	SVM	FFT		0.16	0.33
233	CNN	Time	72/24/24	0.94	0.97
	MobileNet	Time		0.81	0.89
	CNN	FFT		0.91	0.96
	MobileNet	FFT		0.80	0.92
	SVM	FFT		0.77	0.88
007	CNN	Time	46/23/23	0.92	0.96
	MobileNet	Time		0.65	0.73
	CNN	FFT		0.52	0.77
	MobileNet	FFT		0.22	0.35
	SVM	FFT		0.35	0.55
011	CNN	Time	29/23/23	0.83	0.90
	MobileNet	Time		0.72	0.88
	CNN	FFT		0.04	0.11
	MobileNet	FFT		0.07	0.09
	SVM	FFT		0.33	0.61

Train/test/eval counts denote 5 s segments.

PC-F1, positive-class F1; PR-AUC, area under the precision–recall curve.

Across subjects, the time-domain CNN consistently provided the strongest verification performance. Averaged over all five enrolled subjects, the time-domain CNN achieved **0.91** ± **0.05 PC-F1** and **0.96** ± **0.03 PR-AUC** (Table 4). The best result was observed for Subject 180 with **PC-F1** = **0.95** and **PR-AUC** = **0.99**, indicating highly separable per-subject embeddings and a robust precision–recall trade-off. The comparatively lower performance on Subject 011 (**PC-F1** = **0.83**) likely reflects a combination of smaller training set size (29 training segments) and greater intra-recording variability. Notably, the narrow standard deviations for the time-domain CNN contrast with the larger variability of FFT-domain models (e.g., CNN FFT PC-F1 SD = 0.33), suggesting that spectral features are more sensitive to subject- and session-specific conditions.

**Table 4:** Mean ± standard deviation of positive-class F1 (PC-F1) and area under the precision–recall curve (PR-AUC) across all enrolled subjects.

Model	Domain	PC-F1 (mean ± SD)	PR-AUC (mean ± SD)
CNN	FFT	0.51 ± 0.33	0.67 ± 0.33
CNN	Time	0.91 ± 0.05	0.96 ± 0.03
MobileNet	FFT	0.46 ± 0.32	0.56 ± 0.35
MobileNet	Time	0.70 ± 0.18	0.82 ± 0.13
SVM	FFT	0.46 ± 0.26	0.65 ± 0.23

**Table 5:** Quantization and embedded model analysis.

Parameter	Specification
Model format	ONNX
Pre-quantization size	123 KB (float32 model)
Post-quantization size	48 KB (int8 static quantization)
Compression ratio	60.9% reduction in model size
Quantization type	Static Quantization (ONNX → ONNX_Q8)
MACC operations	1,983,138 Multiply-Accumulate Operations
Weights memory (RO)	28,484 B (27.82 KB)
Activation memory (RW)	8,980 B (8.77 KB)
Total RAM usage	8,980 B (includes activation buffer)
Input tensor	int8(1x2500), 2.44 KB, QLinear(0.2578, −50, int8)
Output tensor	int8(1x1), 1 Byte, QLinear(0.0039, −128, int8)

In four out of five subjects, time-domain models clearly outperformed their FFT-based counterparts, consistent with the aggregate statistics in Table 4. An interesting exception is Subject 233, where the FFT-domain CNN reached **PC-F1** = **0.91** and **PR-AUC** = **0.96**, closely trailing the time-domain CNN (0.94/0.97). This suggests that, for certain individuals, frequency-encoded morphology may capture subject-specific traits that are nearly as discriminative as time-local features. Nevertheless, FFT-domain performance was generally weaker (e.g., Subjects 007 and 011), indicating that phase and temporal context remain important for stable verification across diverse recording conditions.

The lightweight time-domain CNN consistently surpassed MobileNet variants on this task. Notably, Subject 093 showed a pronounced gap for MobileNet in the time domain (**PC-F1** = **0.42**), while the same architecture performed substantially better in the FFT domain (0.75). On average (Table 4), MobileNet Time reached **0.70** ± **0.18 PC-F1**, reflecting higher across-subject variability than the CNN Time, whereas MobileNet FFT averaged 0.46 ± 0.32 PC-F1. We hypothesize that depthwise separable convolutions may be more sensitive to per-subject normalization and limited data in the time domain, whereas their inductive bias transfers more gracefully to spectral representations. Classical SVMs in the FFT domain were occasionally competitive (e.g., Subject 180, **PC-F1** = **0.68** vs. MobileNet FFT at 0.44), underscoring that simpler decision boundaries can work reasonably well when features emphasize stable, subject-specific spectral bands.

Performance correlated with the amount and diversity of training material per subject. Subject 180 benefited from the largest pool of recordings, allowing us to reserve multiple unseen records for validation and testing while maintaining a sizable training set. In contrast, Subject 011 exhibited the lowest CNN time-domain PC-F1 (0.83), which we attribute to both fewer training segments and potentially higher within-subject variability. This observation aligns with a broader principle in personalized biometrics: subject-specific models improve with increased coverage of day-to-day variation, electrode placement drift, and noise conditions.

Reported PC-F1 values reflect thresholds selected on per-subject validation sets to optimize F1. The high PR-AUC values (often ≥0.96) indicate that scores are well-calibrated for ranking, offering flexibility to tune operating points post-deployment—for example, targeting low false accept rates in clinical workflows while retaining acceptable true accept rates. In practice, we recommend setting subject-specific thresholds based on a short enrollment calibration to match task requirements (e.g., stricter verification vs. more permissive monitoring).

From an embedded deployment perspective, we find it encouraging that a compact time-domain CNN reliably outperforms heavier or more generic architectures while remaining deployable on a microcontroller. The consistent edge of time-domain features suggests that preserving temporal morphology—even in short, 5-s windows—provides strong identity cues. At the same time, the near-parity of FFT performance for Subject 233 is a useful reminder that some users may present more stable frequency-domain signatures, which could be exploited in hybrid or adaptive pipelines. For the few lower-performing cases, we expect that modest additions—beat-synchronous alignment, rhythm-aware pooling, or subject-tailored data augmentation (e.g., controlled baseline wander, mild heart-rate shifts)—would close much of the gap without sacrificing on-device efficiency.

Figure 4 illustrates the evolution of training and validation loss across 50 epochs. The validation loss exhibited a smooth and steady decline, reaching its lowest value of **0.0289** at epoch **35**, indicating strong generalization ability. The training loss followed a similar downward trajectory.

## 8 Neural network embedded implementation

### 8.1 Model quantization

To deploy the neural network efficiently on the STM32H7 microcontroller, the model underwent a structured quantization pipeline. The objective of this process was to reduce the memory footprint and computational overhead while maintaining classification accuracy. The trained model was first exported from PyTorch format to the ONNX format, ensuring compatibility with embedded AI deployment tools. The ONNX model preprocessing functionality was then used to simulate inference behavior and define the required calibration parameters.

Quantization was performed using static post-training quantization, converting floating-point computations into fixed-point arithmetic. This transformation involved mapping all network operations, including convolutions and fully connected layers, to integer precision (int8). The model was statically quantized to ONNX_Q8 format, applying affine transformations to both activations and weights. This reduced the model size from 123 KB in floating-point precision to 48 KB, achieving a compression ratio of 60.9%. Parameter details of the quantization process are listed in Table 5.

The static quantization process relies on affine integer transformations to scale floating-point values into an 8-bit fixed-point range. Quantization of inputs and outputs follows the standard QLinear transformation described by Equations 1, 2: ?>

$$X_{\text{int8}}=\text{round}\left(\frac{X_{\text{\textbackslash{},float32}}}{S_{X}}\right)+Z_{X}$$ (1)

$$X_{\text{\textbackslash{},float32}}=S_{X}\times (X_{\text{int8}}-Z_{X})$$ (2)

where $X_{\text{\textbackslash{},float32}}$ represents the original floating-point input, $X_{\text{int8}}$ is the quantized integer representation, $S_{X}$ is the scale factor, and $Z_{X}$ is the zero-point offset. This mapping allows all matrix multiplications and convolutions to be performed using integer arithmetic, significantly reducing computational complexity.

Substituting the calibrated parameters into Equations 1, 2, the quantization for the ECG model becomes Equations 3, 4: ?>

$$X_{\text{int8}}=\text{round}\left(\frac{X_{\text{\textbackslash{},float32}}}{0.2578}\right)-50$$ (3)

$$X_{\text{\textbackslash{},float32}}=0.0039\times (X_{\text{int8}}+128)$$ (4)

### 8.2 Quantized model performance

Quantization did not adversely impact the model’s classification performance. The 8-bit integer quantized model achieved accuracy, precision, recall, and F1-score metrics equivalent to those of the original 32-bit floating-point PyTorch model within the expected margin of evaluation variability. These results confirmed that efficient model deployment on resource-constrained embedded hardware was possible without compromising predictive performance.

**Figure 5:**
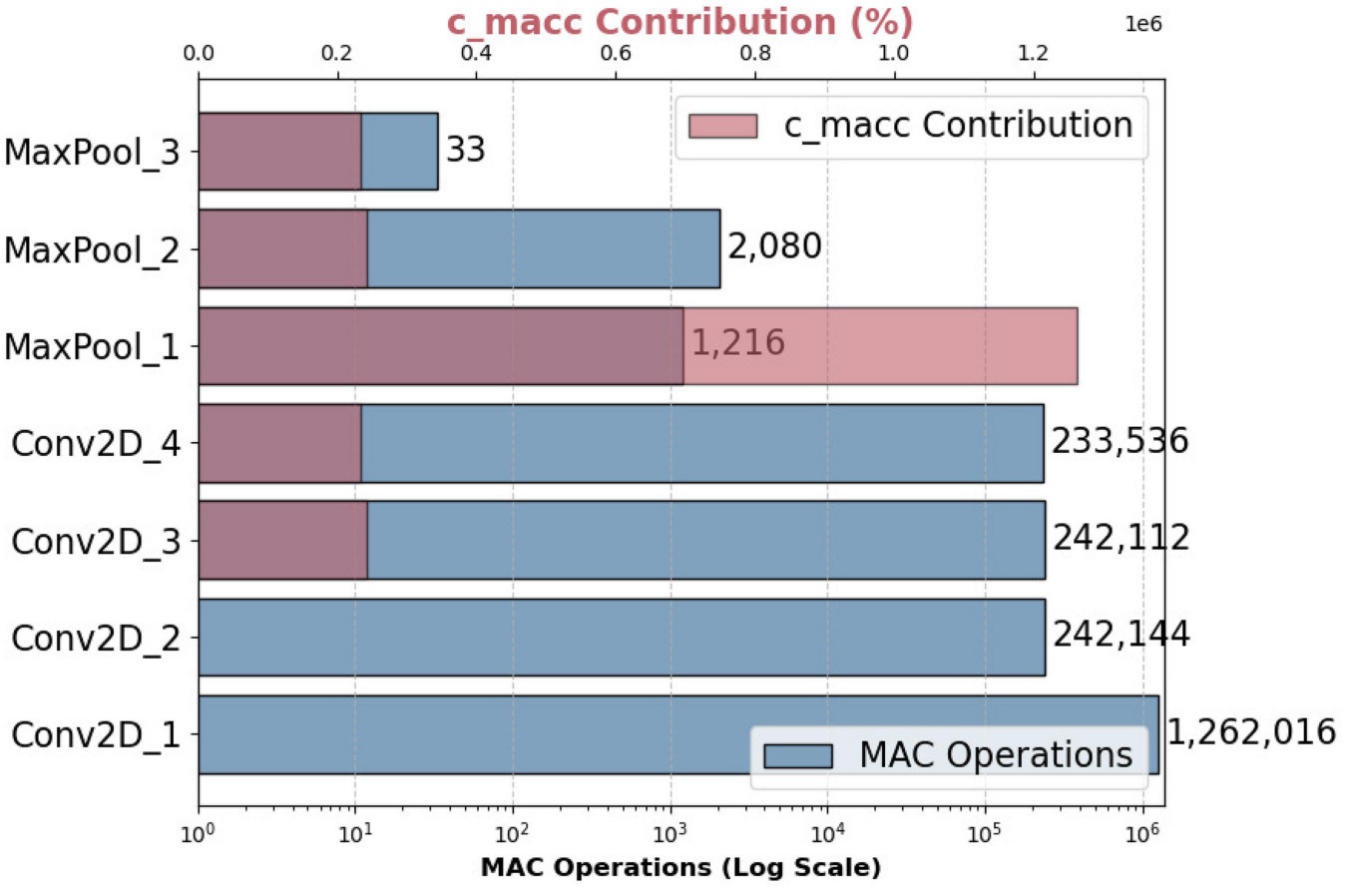
Operation complexity breakdown of the quantized ECG model on STM32H7. This figure presents a layer-wise analysis of computational complexity, comparing the number of Multiply-Accumulate (MAC) operations (blue bars) with the actual computational cost (c_macc contribution, red bars), which accounts for memory access and processing overhead.

As shown in Figure 5, while convolutional layers dominated in raw MAC operations, pooling layers exhibited a disproportionately high actual Multiply-Accumulate (MAC) cost (c_macc), with MaxPool${}_{1}$ alone accounting for 63.6% of total computational complexity despite performing a negligible number of MAC operations. This behavior was attributed to memory-bound inefficiencies, where pooling operations require multiple unoptimized memory fetches, strided indexing, and cache-inefficient comparisons [56]. The dual X-axis visualization distinguished between pure arithmetic intensity (log scale) and real-world execution cost (linear scale), revealing that memory overhead outweighed pure computation in embedded AI inference.

While convolutional layers benefited from SIMD acceleration and optimized matrix-multiplication kernels on the STM32H7 platform [56], pooling operations lacked comparable parallelization support, resulting in execution bottlenecks. These observations were consistent with prior work emphasizing the importance of memory access patterns in constrained environments [56].

Based on these findings, stride-based convolutions or average pooling operations could offer more efficient alternatives to max-pooling in resource-limited deployments, consistent with strategies used in lightweight architectures such as MobileNet [57].

**Figure 6:**
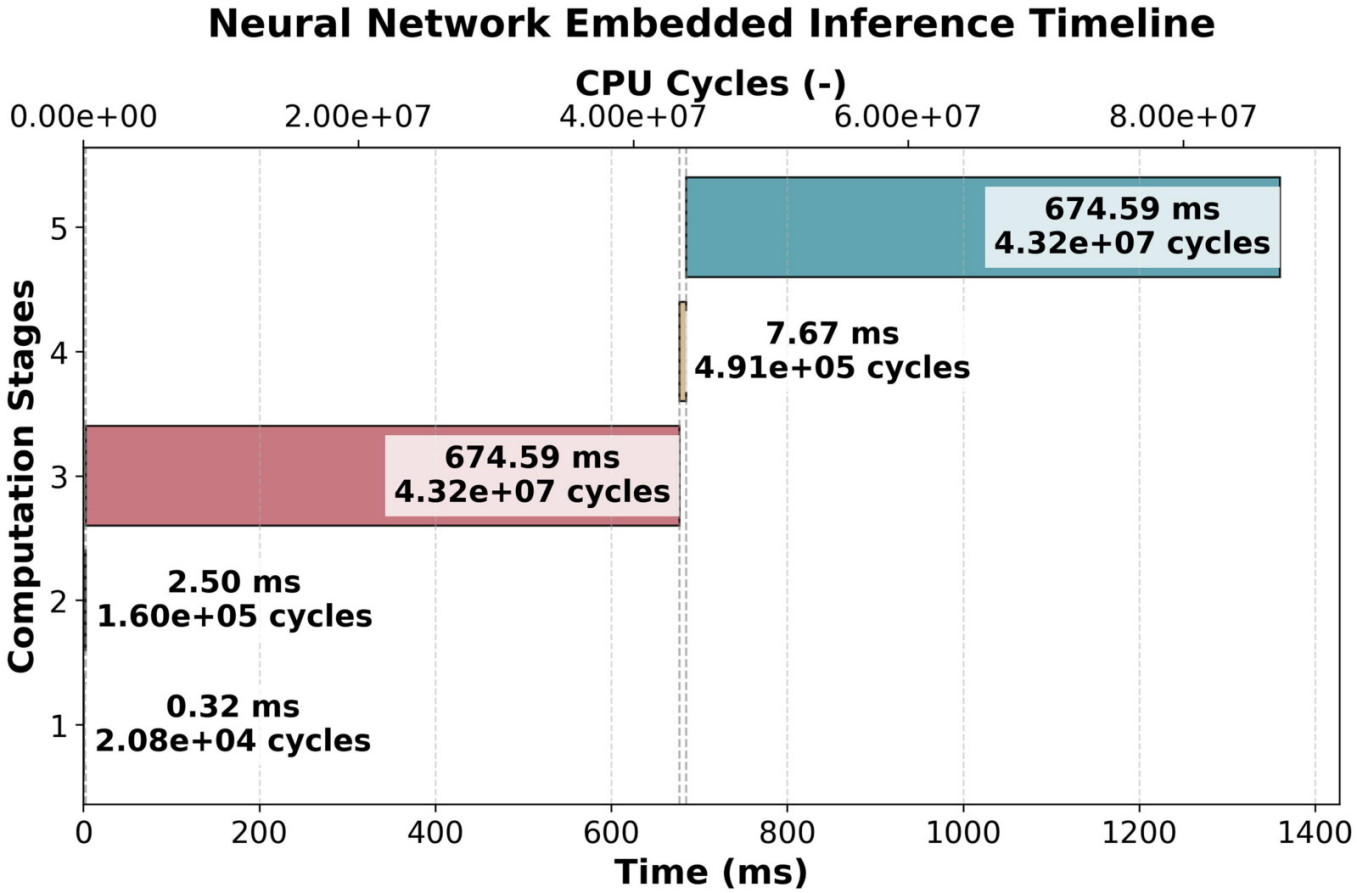
Embedded neural network inference timeline, showing execution stages in both milliseconds (ms) and CPU cycle counts. The processing pipeline is divided into five stages: (1) downsampling, (2) standardization, (3) first inference (signal quality assessment), (4) weights reload, and (5) second inference (biometric classification). The total execution time of approximately 1.35 s ensures real-time processing for 5-s ECG data blocks.

The execution efficiency of deep learning models on embedded systems was evaluated using the timeline shown in Figure 6, which presents execution time across five computational stages, measured in both milliseconds and CPU cycle counts. The stages included downsampling, standardization, first inference, weights reload, and second inference. Analysis of this timeline enabled identification of computational bottlenecks, memory access overhead, and the real-time implications of quantized inference.

The first inference stage, which required 674.59 ms, served as a signal quality assessment step. Based on its output, computation proceeded conditionally. If the signal quality was marked as sufficient for further processing, the system reloaded a new set of model weights (7.67 ms) and performed a second inference (674.58 ms). This conditional execution strategy enabled adaptive processing, allowing the system to adjust computational resources based on signal characteristics.

From a computational perspective, inference remained the most resource-intensive operation. The first and second inference stages together accounted for approximately 98% of the total execution time. Preprocessing steps such as downsampling (0.32 ms) and standardization (2.49 ms) imposed relatively low computational demand, though standardization still introduced measurable overhead due to floating-point arithmetic.

A complementary analysis of CPU cycle counts confirmed the dominance of inference-related operations. Each inference stage required approximately 43,173,585 CPU cycles. The weights reload stage, despite its short execution time, incurred 490,852 CPU cycles due to memory access operations. The standardization step consumed 159,898 cycles, compared to only 20,776 cycles for downsampling, suggesting that preprocessing contributed non-negligibly to overall latency.

Given that the system processed each 5-s ECG block in approximately 1.35 s, the implementation satisfied the real-time constraint of completing inference within the acquisition window. The available processing margin ensured that the system remained responsive and capable of continuous operation, leaving headroom for additional tasks such as data storage or transmission.

## 9 Baseline comparisons

Table 6 summarizes representative studies on ECG-based biometric identification and embedded ECG classification, focusing on reported performance, model size, and computational complexity. Deep learning approaches such as those by Agrawal et al. [39] and Mangold et al. [47] demonstrated the strong discriminative potential of ECG morphology for user identification, achieving accuracies exceeding 98%. However, these works relied on high-capacity neural networks trained and evaluated in GPU or cloud environments, without addressing the constraints of wearable or resource-limited devices.

**Table 6:** Comparison of related ECG-based biometric and embedded learning studies in terms of accuracy, model size, and computational complexity.

Study	Dataset/subjects	Task type	Performance	Model size/parameters	Computational complexity/platform
Agrawal et al. [39]	PTB Diagnostic ECG Database, multi-subject authentication	User authentication	Accuracy 98.34% (CNN), 99.69% (LSTM)	Not reported	Standard GPU inference; cloud environment
Wang et al. [50]	PTB-DB (290 subjects), MIT-BIH, ECG-ID	Authentication (self-supervised)	Accuracy 99.1% (PTB), ≤ 98.5% on external datasets	Not stated; 8-bit quantized/pruned	Cortex-M4F microcontroller; ≈ 37% reduction in compute cost
Mangold et al. [47]	∼100,000 patients, 970,000 ECGs	Biometric identification (Siamese NN)	0.97 AUC	Large-scale model (not specified)	GPU/cloud environment; long-term ECG fingerprints
Raj and Ray [48]	Real-time embedded ECG monitor & MIT-BIH	Arrhythmia detection	Accuracy 96.14%	DSP pipeline & TSVM	Low-power MCU; handcrafted feature extraction
This work	PTB-DB, single-lead (aVL), Subject 180 + 4 others	Personalized biometric verification	94.68% accuracy, F1 = 94.51%, AUC = 0.9986	123 kB (float32) → 48 kB (int8)	1.35 s total inference on STM32H7 (Cortex-M7); real-time on-device

Recent progress has begun to shift toward edge-oriented and lightweight implementations. Wang et al. [50] presented a self-supervised ECG authentication framework optimized for IoT edge sensors, employing model pruning and 8-bit quantization to reduce computational cost by approximately 37% on a Cortex-M4F microcontroller, while maintaining near 99% accuracy on the PTB database. Similarly, Raj and Ray [48] demonstrated real-time ECG processing on a low-power microcontroller using classical DSP and machine learning techniques.

In contrast, the present work represents a fully embedded and personalized ECG biometric system capable of performing both signal-quality assessment and identity verification in real time on an STM32H7 microcontroller. Despite its compact memory footprint of only 48 kB after 8-bit quantization, the proposed convolutional neural network achieved 94.68% classification accuracy and an F1-score of 94.51%, with a total inference time of approximately 1.35 s per 5 s ECG segment.

## 10 Conclusion and future work

This study demonstrated the feasibility of deploying real-time neural network inference for biometric ECG classification directly on a resource-constrained Holter device. By integrating a quantized convolutional neural network onto an STM32H7 microcontroller and employing a two-stage inference pipeline with signal-quality assessment, the system achieves real-time performance with efficient computational resource usage. The implementation confirms that low-power wearable devices can support continuous ECG biometrics with reduced reliance on cloud infrastructure.

While the achieved results validate the technical viability of personalized on-device ECG classification, the findings also highlight the importance of broader data diversity for further refinement. Recordings acquired under similar conditions (e.g., resting or supine posture) may exhibit high morphological consistency, potentially limiting model generalization. Future work will therefore focus on longitudinal data collection across multiple postures, daily activities, and physiological states. This will enable the model to learn invariant, subject-specific cardiac representations and adapt to gradual physiological changes, reducing overfitting while expanding applicability toward early detection of pathological trends. Ultimately, the proposed framework represents a stepping stone toward fully personalized, self-contained, and adaptive cardiac monitoring in everyday life.

## Data Availability

The original contributions presented in the study are included in the article/Supplementary Material, further inquiries can be directed to the corresponding author/s.
